# The efficiency of ultrasound-guided erector spinae plane block in early cervical cancer patients undergoing laparotomic radical hysterectomy: A double-blind randomized controlled trial

**DOI:** 10.3389/fsurg.2022.1039629

**Published:** 2023-01-23

**Authors:** Ling Zhou, Shan Wang, Chunmei Liu, Tingting Yan, Youping Song, Shuhua Shu, Sheng Wang, Xin Wei

**Affiliations:** Department of Anesthesiology, The First Affiliated Hospital of USTC, Division of Life Sciences and Medicine, University of Science and Technology of China, Hefei, China

**Keywords:** TAPB, analgesic effect, opioids consumption, laparotomic radical hysterectomy, ESPB

## Abstract

**Aims:**

We aim to compare the efficiency of erector spinae plane block (ESPB) with transversus abdominis plane block (TAPB) in patients undergoing laparotomic radical hysterectomy because only a few studies are reported exploring this matter.

**Methods:**

In this randomized controlled trail, 154 eligible patients were randomly allocated into ESPB group [ESPB + patient-controlled intravenous analgesia (PCIA)] and TAPB group (TAPB + PCIA) at 1:1 ratio. The primary outcome was visual analog scale (VAS) score at rest state at 12 h.

**Results:**

We found that ESPB group was associated with the lower VAS scores at rest and cough state than TAPB group at 2, 4, 6, 12, and 24 h postoperatively (*P* < 0.05). Less analgesic consumption and sufentanil consumption in PCIA pump were found in the ESPB group (*P* < 0.05). Moreover, ESPB group was followed by fewer rescue analgesia requirements, less rescue analgesic consumption, less adverse reactions, and higher analgesia satisfaction (*P* < 0.05).

**Conclusions:**

Our study found that ESPB had advantages on analgesic effect and opioids consumption. In the future, more studies were needed to confirm our findings.

**Systematic Review Registration:** https://www.chictr.org.cn/index.aspx, identifier: ChiCTR2100044240.

## 1. Introduction

Laparotomic radical hysterectomy is one of the effective methods for women with early cervical cancer, but the postoperative pain management remains unsatisfactory (1). The untreated postoperative pain may delay postoperative recovery, prolong hospitalization, and increase the risk of chronic pain and thromboembolism (2, 3). Patient-controlled intravenous analgesia (PCIA) is commonly used to relieve postoperative pain through intravenous injection of opioids; however, opioids have drug-addiction and produce adverse reactions, such as nausea, vomiting, constipation, and respiratory depression (4). Therefore, it needs an active exploration to implement effective nonopioid or opioid-reduced pain management strategies.

Multimodal analgesia refers to the simultaneous use of several different analgesic drugs or techniques to provide opioid-reduced or opioid-free anesthesia, with regional anesthesia at its core (5). Accordingly, it is a focus to investigate the ability of regional anesthesia for postoperative pain management in recent years (5). Transversus abdominis plane block (TAPB) has been widely used in hysterectomy (6, 7). Compared with placebo or no block, TAPB effectively controlled the early and delayed pain, and reduced the consumption of opioids in patients undergoing laparotomic radical hysterectomy (6). In addition, TAPB combined with PCIA lowered the pain level within 24 h after laparotomic radical hysterectomy and prolonged the time to first analgesic requirement (7). The main sources of pain of patients undergoing abdominal surgery are the anterior abdominal wall and abdominal viscera (8). Although TAPB displayed good effect on somatic pain, it failed to effect on visceral nerves (9). In 2016, Forero et al. first reported erector spinae plane block (ESPB), which may not only reduce somatic pain but also improve visceral pain since it blocked the ventral, dorsal, and communicating branches of the spinal nerve (10). Compared with placebo, ESPB had advantages in the use of opioids and postoperative pain after laparotomic radical hysterectomy (11). A randomized controlled trial (RCT) of 48 patients showed that ESPB provided potent postoperative analgesia with less consumption of opioids than TAPB for patients undergoing laparotomic radical hysterectomy (12). Due to studies comparing the analgesia and opioids consumption between TAPB and ESPB were limited and the sample size was small, further explorations were needed.

Herein, we aimed to design an RCT to compare the efficiency of TAPB and ESPB based on a larger sample size to evaluate the application value of ESPB in postoperative analgesia of patients undergoing laparotomic radical hysterectomy.

## 2. Methods

### 2.1. Study design

This double-blinded RCT had obtained an approval from the Ethics Committee of the First Affiliated Hospital of USTC, Division of Life Sciences and Medicine, University of Science and Technology of China (approval number: 2021KY-020), and all patients had provided the written informed consent. This trial was carried out according to the principles outlined in the Declaration of Helsinki, and had registered in Chinese Clinical Trial Registry (registration number: ChiCTR2100044240).

### 2.2. Participants

The patients were recruited in the First Affiliated Hospital of USTC, Division of Life Sciences and Medicine, University of Science and Technology of China from March 2021 to November 2021. The women who aged ≥18 years, with early cervical cancer [International Federation of Gynecology and Obstetrics (FIGO) stage of IA–IIA], scheduled to undergo laparotomic radical hysterectomy and required for postoperative analgesia, with an American Society of Anesthesiology (ASA) physical status of I–II, without communication barriers (had abilities to implement the trial, to understand the use of relevant scales, and to operate PCIA equipment), voluntarily participated and signed the informed consent were included. The patients who met one of the following criteria were excluded: (1) with local infection at puncture site; (2) with severe hepatic and kidney impairment and hematologic disorders (including coagulation abnormality); (3) with history of abdominal surgeries or abdominal trauma; (4) using sedative and analgesic drugs for a long term or addicting to alcohol, sedative, and analgesic drug; (5) with chronic pain; (6) allergy to drugs used in this study; (7) with mental illness that interfered perception and pain assessment; (8) pregnant or lactating women; (9) complicated with diffuse peritonitis, umbilical hernia, diaphragmatic hernia, abdominal wall hernia, inguinal hernia, or femoral hernia; and (10) participating in other clinical trials within 30 days.

### 2.3. Randomization and blinding

Patients were randomly divided into TAPB group (TAPB combined with PCIA) and ESPB group (ESPB combined with PCIA) at 1:1 ratio according to the computer-generated sequence numbers, which were hid through opaque sealed envelopes. A designated person kept the random code table listing the treatment allocation corresponding to the serial number of 001–154, and the serial number corresponded to the number of patients. After patients were included, the researcher informed the keeper of the corresponding patient number, and the keeper gave the instruction of the patient entering the TAPB group or ESPB group according to the random code table. After receiving this instruction, the researcher made corresponding records and implemented corresponding allocations. To make patients be blinded, blocks were performed just before extubation. Also, efficacy assessor was blinded to the group assignment.

### 2.4. Intervention

Before the operation, cardiopulmonary function and anesthesia risk of patients were assessed, and nutritional screening and nutritional support were given to the patients. The patients without gastrointestinal dysfunction were forbidden to eat at preoperative 6 h and to drink at preoperative 2 h. After patients entering the operating room, upper extremity venous access was developed routinely, and blood pressure (BP), heart rate (HR), electrocardiogram (ECG), and blood oxygen saturation (SpO_2_) were monitored. Midazolam (0.03–0.05 mg/kg), sufentanil (0.3–0.4 μg/kg), etomidate (0.2–0.3 mg/kg), and rocuronium (0.6–0.8 mg/kg) were given for anesthesia induction. Satisfied with the anesthesia induction, endotracheal intubation and mechanical ventilation were performed. The anesthesia was maintained by combined intravenous-inhalation anesthesia. The intravenous anesthesia was performed by intravenous infusion of propofol [4–6 mg/(kg h)] and remifentanil [6–10 μg/(kg h)]. The drug used for inhalation anesthesia was sevoflurane, and the concentration was adjusted according to patient's hemodynamics. Cisatracurium was intermittently injected to maintain muscle relaxation.

After suturing the surgical incision, ESPB or TAPB was immediately performed on the patients by the same anesthesiologist. After extubation, patients in both groups were given PCIA pump (100 ml) with ingredients of sufentanil (100 μg), flurbiprofen axetil (100 mg), ondansetron (16 mg), and 0.9% sodium chloride injection. PCIA pump is set with background dose of 2 ml/h, and patient-controlled dose was 2 ml/time, with lockout time of 30 min. Single intravenous injection of flurbiprofen axetil was given as rescue analgesia for patients at rest with visual analog scale (VAS) score ≥ 4. The rescue analgesia requirement and time to the first rescue analgesia were recorded. The follow-up at 2, 4, 6, 12, and 24 h postoperatively was completed by the ward nurses and the anesthesia nurses.

#### 2.4.1. ESPB group

Patients were placed in the lateral decubitus, and ESPB was performed at the level of the nine thoracic vertebrae (T9). The linear high-frequency array probe of color two-dimensional ultrasound instrument (Navis, Wisonic, Shenzhen, China) was used for sagittal scanning, and placed sagittal 3 cm lateral to T9 transverse process. 20 ml of injection containing 0.375% ropivacaine was injected at one time between the deep surface of erector spinae on the upper or lower sides and the T9 transverse process using in-plane needle insertion, and a total of 40 ml injection was injected on both sides. Ultrasound confirmation of the local anesthetic spread was seen as an anechoic shadow in the paravertebral space between T7 and T12.

#### 2.4.2. TAPB group

Patients were placed in the supine position, and petit triangle was first determined and marked. The ultrasound probe was placed in the region between costal margin and anterior superior spine and paralleling to anterior axillary line to scan. The part with clear anatomical structure of abdominal wall muscle layer was selected, and then the needle was entered under the guidance of ultrasound until the tip of puncture needle entered into the plane between the rectus abdominis and the internal oblique. After the puncture needle was drawn back and no blood was found, 20 ml of injection containing 0.375% ropivacaine was slowly injected into the plane, and a total of 40 ml injection was injected on both sides. An expanding hypo-echoic area was seen in the transversus abdominis plane under the ultrasound.

### 2.5. Outcomes

#### 2.5.1. Primary outcome

The primary outcome was VAS score at rest state at postoperative 12 h.

The 100 mm VAS was used to measure pain intensity, ranging from 0 (no pain) to 100 (severe pain) (13).

#### 2.5.2. Secondary outcomes

The secondary outcomes were VAS score at rest state at postoperative 2, 4, 6, and 24 h; VAS score at cough state at postoperative 2, 4, 6, 12, and 24 h; press times of PCIA pump at postoperative 2, 4, 6, 12, and 24 h; analgesic consumption in PCIA pump at postoperative 2, 4, 6, 12, and 24 h; sufentanil consumption in PCIA pump at 2, 4, 6, 12, and 24 h; Ramsay sedation scale (RSS) at postoperative 2, 4, 6, 12, and 24 h; operation time; intraoperative bleeding; first time to press PCIA pump; first time out of the bed; first time to exhaust; time to remove urinary catheter; hospital stay; analgesia satisfaction; rescue analgesia requirement; rescue analgesic consumption at 24 h; and adverse reactions.

The dose of analgesic consumption in PCIA pump was obtained according to the reading of PCIA. Sufentanil consumption in PCIA pump was calculated based on sufentanil concentration (1 μg/ml) and analgesic consumption in PCIA pump.

RSS was used to assess patients’ levels of sedation (14). Patients scored 1 point were anxious, agitated, and restless; scored 2 points were cooperative, oriented, and tranquil; scored 3 points had response to commands; scored 4 points had brisk response to stimulus; scored 5 points had sluggish response to stimulus; and scored 6 points had no response to stimulus (14).

Analgesia satisfaction was assessed using 4-point Likert scale, with 1 point representing very dissatisfied, 2 points representing somewhat dissatisfied, 3 points representing somewhat satisfied, and 4 points representing very satisfied (15, 16).

Adverse reactions included nausea, vomiting, cough, and fever.

### 2.6. Sample size

The sample size was calculated based on the VAS score at rest state at 12 h after the operation, and the formula was shown as follows:

$$N1=N2_{\;}=2{\left[\frac{\left(u_{\alpha }+u_{\beta }\right)}{\delta /\sigma }\right]}^{2}+u_{\alpha }^{2}/4$$

The bilateral *α* was 0.05, and $u_{0.05/2}$ was 1.96; *β* was 0.1, and $u_{0.01}$ was 1.282. According to the published study (11), VAS score at rest state at postoperative 12 h in the ESPB group was predicted to be 4.5 ± 1.54, and that in the TAPB group was predicted to be 5.2 ± 0.76, then *δ* = 0.7, *σ* = 1.21. We calculated that 63 patients were needed in each group. Considering a dropout rate of 15%, the final sample size required for a single group is 75, and a total of 150 patients were needed.

### 2.7. Statistical analysis

Kolmogorov–Smirnov test was used to test the normality of measurement data. The measurement data in normal distribution were described as mean ± standard deviation (mean ± SD), and comparison between the two groups was implemented using independent sample *t*-test. The measurement data in non-normal distribution were described as median and interquartile range [M (Q1, Q3)], and rank-sum test was used for comparison between groups. The counting data were described as number and percentage [*n* (%)], and *χ*^2^ test was used for comparison between groups. The ranking data was shown as *n* (%), and compared using rank-sum test. All statistical tests were performed using SAS 9.4 (SAS Institute Inc., Cary, NC, Unites States) with two-sided test, and *α* = 0.05. The stacking chart, bar chart, and box chart were drawn using Python 3.9.7 (Python Software Foundation, DE, United States). *P* < 0.05 was considered to be statistically significant.

## 3. Results

### 3.1. Comparison of general information between ESPB and TAPB groups

**Figure 1:**
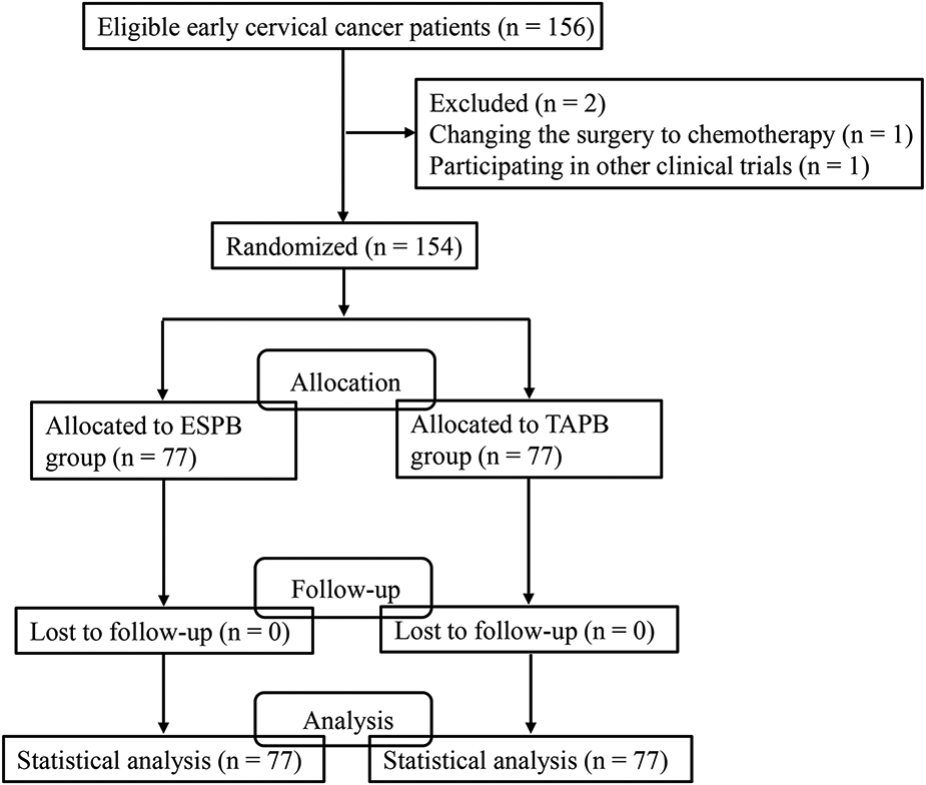
The flowchart of patient selection.

Figure 1 shows that a total of 156 eligible early cervical cancer patients were enrolled in this study. Two patients were excluded because one of them changed the surgery to chemotherapy and one of them participated in other clinical trials. Finally, 154 patients were randomly allocated into ESPB group (*n* = 77) and TAPB group (*n* = 77). No one was lost during the follow-up; therefore, 77 patients in the ESPB group and 77 patients in the TAPB group were included for statistical analysis. The two groups were comparable regarding age, height, weight, body mass index (BMI), FIGO stage, and ASA physical status. There was no significant difference with regard to these parameters between the two groups (Table 1).

**Table 1:** Comparison of general information of erector spinae plane block group and transversus abdominis plane block group.

Variables	Total (*n* = 154)	ESPB (*n* = 77)	TAPB (*n* = 77)	*P*
Age, year	50.47 ± 9.07	49.99 ± 9.48	50.95 ± 8.68	0.513
Height, m	1.60 ± 0.05	1.60 ± 0.05	1.59 ± 0.05	0.498
Weight, kg	60.42 ± 8.70	59.90 ± 8.45	60.94 ± 8.96	0.463
BMI, kg/m^2^	23.70 ± 3.13	23.40 ± 2.96	24.00 ± 3.29	0.237
FIGO stage				0.481
IA	16 (10.39)	8 (10.39)	8 (10.39)	
IB	106 (68.83)	50 (64.94)	56 (72.73)	
IIA	32 (20.78)	19 (24.68)	13 (16.88)	
**ASA status**				
II	154 (100.00)	77 (100.00)	77 (100.00)	

ESPB, erector spinae plane block; TAPB, transversus abdominis plane block; BMI, body mass index; FIGO, international federation of gynecology and obstetrics; ASA, American society of anesthesiology; mean ± SD, mean ± standard deviation.

The measurement data in normal distribution were described as mean ± SD, compared using independent sample *t*-test. The counting data were described as n (%), and compared using χ^2^ test.

### 3.2. Comparison of postoperative analgesia and opioids consumption between ESPB and TAPB groups

VAS score at rest were significantly lower in the ESPB group compared with the TAPB group at 2, 4, 6, 12, and 24 h (all *P* < 0.05). Also, ESPB group showed significantly lower VAS score at cough than TAPB group at 2, 4, 6, and 12 h (all *P* < 0.05), while no significant difference was found between the two groups at 24 h. The press times of PCIA pump was not significant at 2, 4, and 6 h between the two groups; however, it was significantly fewer in the ESPB group than the TAPB group at 12 and 24 h. There was statistical significance in the analgesic and sufentanil consumption in PCIA pump between the two groups at 4, 6, 12, and 24 h, with ESPB group had less consumption of both analgesic and sufentanil at each timepoints (all *P* < 0.05). RSS score was not significantly different between the two groups at each timepoint. All results were shown in Table 2.

**Table 2:** Comparison of postoperative analgesia and opioids consumption between the two groups at observation time points after the operation.

Variables	Total (*n* = 154)	ESPB (*n* = 77)	TAPB (*n* = 77)	*P*
**VAS score at rest**				
2 h	1.00 (1.00, 1.00)	1.00 (0.00, 1.00)	1.00 (1.00, 2.00)	0.003
4 h	2.00 (1.00, 2.00)	1.00 (1.00, 2.00)	2.00 (1.00, 2.00)	<0.001
6 h	2.00 (1.00, 2.00)	2.00 (1.00, 2.00)	2.00 (2.00, 3.00)	<0.001
12 h	2.49 ± 0.78	2.27 ± 0.66	2.71 ± 0.82	<0.001
24 h	2.00 (2.00, 2.00)	2.00 (1.00, 2.00)	2.00 (2.00, 2.00)	0.012
**VAS score at cough**				
2 h	2.00 (2.00, 3.00)	2.00 (1.00, 2.00)	2.00 (2.00, 3.00)	0.003
4 h	2.57 ± 0.72	2.38 ± 0.74	2.77 ± 0.65	<0.001
6 h	3.01 ± 0.81	2.82 ± 0.82	3.19 ± 0.76	0.004
12 h	3.56 ± 0.77	3.35 ± 0.66	3.77 ± 0.81	<0.001
24 h	2.93 ± 0.66	2.84 ± 0.76	3.01 ± 0.53	0.112
**Press times of PCIA pump**				
2 h	0.00 (0.00, 0.00)	0.00 (0.00, 0.00)	0.00 (0.00, 0.00)	0.083
4 h	0.00 (0.00, 0.00)	0.00 (0.00, 0.00)	0.00 (0.00, 0.00)	0.601
6 h	0.00 (0.00, 1.00)	0.00 (0.00, 0.00)	0.00 (0.00, 1.00)	0.081
12 h	1.00 (1.00, 2.00)	1.00 (0.00, 1.00)	2.00 (1.00, 2.00)	<0.001
24 h	2.00 (1.00, 3.00)	1.00 (1.00, 2.00)	2.00 (2.00, 3.00)	<0.001
**Analgesic consumption in PCIA pump, ml**				
2 h	4.95 ± 0.72	4.95 ± 0.78	4.95 ± 0.67	1.000
4 h	9.56 ± 1.08	9.36 ± 1.15	9.75 ± 0.98	0.025
6 h	14.06 ± 1.44	13.78 ± 1.35	14.35 ± 1.48	0.013
12 h	28.05 ± 3.16	27.14 ± 3.23	28.96 ± 2.84	<0.001
24 h	53.01 ± 3.48	52.30 ± 4.14	53.71 ± 2.49	0.011
**Sufentanil consumption in PCIA pump, μg**				
2 h	4.95 ± 0.72	4.95 ± 0.78	4.95 ± 0.67	1.000
4 h	9.56 ± 1.08	9.36 ± 1.15	9.75 ± 0.98	0.025
6 h	14.06 ± 1.44	13.78 ± 1.35	14.35 ± 1.48	0.013
12 h	28.05 ± 3.16	27.14 ± 3.23	28.96 ± 2.84	<0.001
24 h	53.01 ± 3.48	52.30 ± 4.14	53.71 ± 2.49	0.011
**RSS score, points**				
2 h				0.566
2	151 (98.05)	75 (97.40)	76 (98.70)	* *
3	3 (1.95)	2 (2.60)	1 (1.30)	* *
4 h				0.566
2	151 (98.05)	76 (98.70)	75 (97.40)	* *
3	3 (1.95)	1 (1.30)	2 (2.60)	* *
6 h				1.000
2	154 (100.00)	77 (100.00)	77 (100.00)	* *
12 h				1.000
2	154 (100.00)	77 (100.00)	77 (100.00)	* *
24 h				1.000
2	154 (100.00)	77 (100.00)	77 (100.00)	* *

ESPB, erector spinae plane block; TAPB, transversus abdominis plane block; VAS, visual analog scale; PCIA, patient-controlled intravenous analgesia; RSS, Ramsay sedation scale.

The measurement data in normal distribution were described as mean ± SD, and compared using independent sample *t*-test. The measurement data in non-normal distribution were described as M (Q1, Q3), and compared using rank-sum test. The counting data were described as n (%), and compared using χ^2^ test. The ranking data was shown as n (%), and compared using rank-sum test.

### 3.3. Comparison of postoperative recovery, rescue analgesia, and adverse reactions between ESPB and TAPB groups

Compared with the TAPB group, more patients with analgesia satisfaction of 4 points was found in the ESPB group (51.95% vs. 9.09%) (*P* < 0.001). Moreover, patients in the ESPB group requiring rescue analgesia were fewer (11.69% vs. 25.97%), and they consumed less rescue analgesic at 24 h (*P* = 0.032). In addition, no difference was found in the operation time, intraoperative bleeding, and first time to press PCIA pump between the two groups (*P* > 0.05). Regarding to postoperative recovery, first time out of the bed, first time to exhaust, time to remove urinary catheter, and hospital stay were not significantly different between the two groups (*P* > 0.05).

A total of 42 patients had adverse reactions, with 10 patients in the ESPB group and 32 patients in the TAPB group, and statistical difference in adverse reactions was found between the two groups (*P* < 0.001). Moreover, the number of patients occurring nausea and vomiting was significantly fewer in the ESPB group than the TAPB group (11.69% vs. 40.26%) (*P* < 0.001). No difference regarding to other adverse reaction between the two groups were noted. All results were shown in Table 3.

**Table 3:** Comparison of postoperative recovery, rescue analgesia, and adverse reactions between the two groups.

Variables	Total (*n* = 154)	ESPB (*n* = 77)	TAPB (*n* = 77)	*P*
Operation time, min	155.00 (120.00, 215.00)	150.00 (120.00, 200.00)	155.00 (125.00, 225.00)	0.395
Intraoperative bleeding, ml	200.00 (100.00, 300.00)	200.00 (100.00, 200.00)	150.00 (100.00, 300.00)	0.846
First time to press PCIA pump, min	405.00 (300.00, 527.00)	413.00 (248.00, 604.00)	384.00 (305.00, 447.00)	0.347
First time out of the bed, h	23.00 (22.00, 26.00)	23.00 (22.00, 27.00)	23.00 (22.00, 26.00)	0.565
First time to exhaust, h	46.30 ± 13.25	47.56 ± 13.77	45.05 ± 12.68	0.243
Time to remove urinary catheter, h	20.99 ± 0.08	21.00 ± 0.00	20.99 ± 0.11	0.320
Hospital stay, day	7.00 (6.00, 8.00)	7.00 (6.00, 7.00)	7.00 (6.00, 8.00)	0.652
Analgesia satisfaction				<0.001
3	107 (69.48)	37 (48.05)	70 (90.91)	
4	47 (30.52)	40 (51.95)	7 (9.09)	
Rescue analgesia requirements				0.023
No	125 (81.17)	68 (88.31)	57 (74.03)	
Yes	29 (18.83)	9 (11.69)	20 (25.97)	
Rescue analgesic consumption at 24 h, mg	0.00 (0.00, 0.00)	0.00 (0.00, 0.00)	0.00 (0.00, 50.00)	0.032
Total adverse reaction				<0.001
No	112 (72.73)	67 (87.01)	45 (58.44)	
Yes	42 (27.27)	10 (12.99)	32 (41.56)	
Nausea and vomiting				<0.001
No	114 (74.03)	68 (88.31)	46 (59.74)	
Yes	40 (25.97)	9 (11.69)	31 (40.26)	
Other adverse reaction				1.000
No	151 (98.05)	76 (98.70)	75 (97.40)	
Yes	3 (1.95)	1 (1.30)	2 (2.60)	

ESPB, erector spinae plane block; TAPB, transversus abdominis plane block; PCIA, patient-controlled intravenous analgesia.

total adverse reaction includes nausea, vomiting, cough, and fever. Other adverse reaction means cough and fever.

The measurement data in normal distribution were described as mean ± SD, and compared using independent sample *t*-test. The measurement data in non-normal distribution were described as M (Q1, Q3), and compared using rank-sum test. The counting data were described as n (%), and compared using χ^2^ test. The ranking data was shown as n (%), and compared using rank-sum test.

## 4. Discussion

In this study, we found ESPB was associated with the lower VAS scores of patients at rest or cough state, and followed by the reduced analgesic and sufentanil consumption in PCIA pump compared with TAPB. Also, ESPB was associated with less rescue analgesia requirement and rescue analgesic consumption, and came along with less adverse reactions, especially nausea and vomiting.

The postoperative pain management after laparotomic radical hysterectomy was a major concern for clinicians (1). Ultrasound-guided TAPB is a simple technique that reduces postoperative pain and opioids consumption; however, it fails to relieve visceral pain and limits the spread of local anesthetics (9). Ultrasound-guided ESPB is considered an alternative to provide effective postoperative analgesia for abdominal surgery (9, 17). ESPB improves somatic and visceral pain *via* influencing the ventral ramus and rami communicates that contain sympathetic nerve fibers when local anesthetic spreads through the paravertebral space (10, 18, 19). Ropivacaine is a long-term local anesthetic, which can inhibit action potential generation of nerve fiber cell membrane and block the transmission of pain to the central nervous system (20, 21). In this study, we performed ESPB in Chinese patients with ropivacaine (22), and results showed that VAS scores of patients at rest or cough state were lowered in that ESPB group than the TAPB group. Our findings were similar with the previously reported studies (11, 12). Hamed et al. have reported that VAS pain score of patients undergoing laparotomic radical hysterectomy in the first postoperative 12 h was significantly lower in the ESPB group than the control group (11). Kamel et al. has reported that VAS score at the first 24 h after the operation was significantly lower in the ESPB group than the TAPB group (12). We also found less rescue analgesia requirement and rescue analgesic consumption in the ESPB group. The similar finding was observed in the study of Abdelrazik et al. (23) Additionally, in ambulatory surgery, sufficient postoperative analgesia increased patient's satisfaction (24). Herein, we found that patients undergoing laparotomic radical hysterectomy were more satisfied with ESPB than TAPB, which was consistent with the study reported by Kamel et al. (12).

The goals of multimodal analgesia were not only to provide sufficient analgesia, but also to minimize opioids consumption to reduce the adverse reactions (4, 5). Hamed et al. stated that ESPB was associated with the reduced fentanyl consumption in the first 24 h after laparotomic radical hysterectomy (11). Altıparmak et al. found that ESPB reduced >30% of postoperative tramadol consumption compared with TAPB after abdominal surgery (9). Gürkan et al. reported that ESPB decreased morphine consumption more effectively than the control group (5.6 ± 3.43 vs. 4.92 ± 7.44 mg) (25). In agreement with previous studies, our study found that sufentanil consumption in ESPB group was less than TAPB group at the first 24 h postoperatively. Postoperative nausea and vomiting were the common problems and were adverse reactions to opioids (26). A meta-analysis has reported that the use of ESPB reduced 68% of risk of postoperative nausea and vomiting in thoracolumbar spinal surgery (27). In our study, 42 patients had adverse reactions; of these, 40 patients had nausea and vomiting, and the number of patients with nausea and vomiting was less in the ESPB group and statistically significant compared with the TAPB group, indicating that ESPB was associated with the reduced opioids consumption and decreased adverse reactions.

There are several advantages of our study. First, this is a randomized, controlled, double-blind trial, which reduces the selective bias and the bias in evaluating efficiency. Second, our study includes a relatively larger sample size, which makes the results more reliable. Results of this study showed that ESPB was associated with better analgesic effect, less opioids consumption, and less occurrence of adverse reactions. Our findings indicated that ESPB had advantages on analgesia and opioids consumption in patients undergoing laparotomic radical hysterectomy, which provided further reference for the use of ESPB in the laparotomic radical hysterectomy in the clinic.

Also, there are some limitations in this study. First, as a single-center trial, our findings may not be generalized to other populations outside of China. In the future, multicenter studies are needed to verify our findings. Second, subjects included in this study are all patients with early cervical cancer. The analgesic effect of ESPB on other indications of transabdominal surgery needs to further study. Third, the follow-up time is short in our study. The long-term effect of ESPB on analgesia and postoperative recovery should further explore. Fourth, our study uses TAPB as the control, and does not compare the analgesic effect of ESPB with other regional anesthesia blocks. In the future, more studies should be conducted to compare the analgesic effect and opioids consumption of ESPB with TAPB and other regional anesthesia methods in patients undergoing laparotomic radical hysterectomy.

## 5. Conclusion

Our study found the better analgesic effect and less opioids consumption of ESPB than TAPB in the patients undergoing laparotomic radical hysterectomy. Considering few studies regarding ESPB were reported in laparotomic radical hysterectomy, whether ESPB was superior to TAPB and could be popularized to use in clinic needed to further explore.

## Data Availability

The raw data supporting the conclusions of this article will be made available by the authors, without undue reservation.
